# Barriers to Accessing Medicines among Syrian Asylum Seekers and Refugees in a German Federal State

**DOI:** 10.3390/ijerph18020519

**Published:** 2021-01-10

**Authors:** Saleh Aljadeeah, Veronika J. Wirtz, Eckhard Nagel

**Affiliations:** 1 Institute of Medical Management and Health Sciences, University of Bayreuth, Prieserstr. 2, 95440 Bayreuth, Germany; eckhard.nagel@uni-bayreuth.de; 2 Department of Global Health, Boston University School of Public Health, Boston, MA 02118, USA; vwirtz@bu.edu

**Keywords:** asylum seekers and refugees, Syria, Germany, North Rhine-Westphalia, access, barriers, language, medicine, acceptance, alcohol, pork products

## Abstract

In Germany, asylum seekers and refugees (AS&Rs) face challenges when accessing healthcare services including medicines. The aim of this study was to explore the barriers to accessing medicines among Syrian AS&Rs in the state of North Rheine-Westphalia, and to provide an understanding of their perspectives towards taking medicines that contain alcohol or pork products. This study is based on a cross-sectional survey using a combination of sampling methods. We used descriptive statistics to analyze quantitative data. Participants’ answers to an open-ended question yielded qualitative data that were categorized based on the thematic areas they discussed or addressed. Among the 1641 respondents, language barriers had more of an effect on the access to medicines than any other factor studied. The effect of language barriers on access to medicines was more pronounced for female participants, those who were older than 50 years, and participants who had chronic diseases in comparison to the other groups of participants. Male participants and those younger than 50 years of age showed more acceptance towards taking medicines that contain alcohol or pork products. Based on our results, we recommend providing more support for AS&Rs to learn the German language, particularly for female refugees, older refugees, and those with chronic diseases or disabilities. We also recommend providing translated medical leaflets for patients who wish to receive them in their native language. Healthcare providers should try to consider the special conditions of AS&Rs patients and take into account their perspectives about treatments and diseases.

## 1. Introduction

War, conflict, human rights violations, and disasters worldwide forced 79.5 million people to flee their homes by the end of 2019. This is the highest number ever recorded for displaced populations. Globally, Syrians form the largest group of refugees (6.7 million). While the majority of refugees (83%) live in countries that are neighbors to their countries of origin, smaller numbers can reach Europe [1]. Germany hosts the largest group of refugees in Europe (1.5 million refugees in total), with Syrian asylum seekers and refugees (AS&Rs) constituting the largest group of the total AS&Rs population in Germany (42%) [1,2]. This number is low considering the global trend of forced migration or in comparison to the number of hosted refugees in countries such as Turkey (3.6 million). However, since the peak of migration in 2015, the term ‘refugee crisis’ has frequently been in the public discourse, and the issue of migration has become an essential part of political debates both in Germany and other European countries [3,4].

An asylum seeker is: “an individual who is seeking international protection and whose claim has not yet been finally decided on by the country in which the claim is submitted” [5]. A refugee, according to the Geneva 1951 refugee convention, is: “someone who is unable or unwilling to return to their country of origin owing to a well-founded fear of being persecuted for reasons of race, religion, nationality, membership of a particular social group, or political opinion” [6].

The rapid increase in the number of refugees arriving in Germany has posed challenges to public health authorities who provide for the health needs of this group [7]. Access to healthcare services among asylum seekers in Germany is restricted. Articles 4 and 6 of the asylum seekers benefit act (*Asylbewerberleistungsgesetz*, *AsylbLG*) of 1993 state that asylum seekers are entitled to emergency medical care, treatment for acute and painful conditions, care during pregnancy and childbirth, vaccinations and other “necessary preventive measures”. Asylum seekers are also entitled to additional care in cases that could be evaluated or approved to be “essential” for the preservation of health [8]. Once asylum seekers receive a refugee status, they can obtain regular access to health care through normal statutory health insurance. Since March 2015, asylum seekers have been able to regularly access healthcare services after waiting 15 months rather than 48 months [8]. However, even after this improvement in the asylum-seekers benefit act, the restriction to healthcare has been described as *untenable* [9]. Bozorgmehr et al. has argued that restricting the healthcare access of any population group based on their residency status can be regarded as a violation of the right to health [10].

In Germany, there is a co-payment for prescription medicines. Statutory insured persons pay ten percent of the sales price per package for each prescription medicine, up to a maximum of ten euros (about USD12) and a minimum of five euros (about USD6). Children under the age of 18 are exempt from all co-payments. Over-the-counter (OTC) medicines are usually not reimbursed by statutory health insurance. However, this does not apply to children under the age of twelve and adolescents, up to the age of 18, with developmental disorders. For these groups, OTC medicine prescribed by a doctor will be reimbursed [11]. These regulations also apply to refugees and asylum seekers who stay in Germany for more than 15 months. Asylum seekers who stay in Germany for less than 15 months are exempt from co-payments for medicines. However, these medicines need to be prescribed for acute and painful conditions, or for the recovery of illnesses [12]. In 2016, Germany spent more on medicines on a per capita basis (EUR 572) compared to other European Union member states [13]. Expenditures for medicines among asylum seekers in Germany in 2016 were approximately half of what spent on a matched group of statutory insured people [14].

The legal restrictions to accessing healthcare among asylum seekers are also coupled with administrative barriers in some parts of Germany [8]. Before 2014, asylum seekers received quarterly renewable healthcare vouchers, which could be used for doctor’s visits. However, they needed to make a personal request at a social welfare office to obtain these vouchers [15]. This procedure has been criticized, as social-welfare-office employees do not typically have the medical qualifications to make decisions regarding the need for medical treatment [16]. Starting from 2015, each federal state of Germany was able to decide whether to keep the healthcare vouchers for asylum seekers or grant them electronic health insurance cards from the beginning of their stay. The majority of German federal states decided to introduce electronic health insurance cards, with the exception of the states of Saxony, Bavaria, and some municipalities in North Rhine-Westphalia (NRW) [15,17,18].

In addition to the system-related restrictions or administrative barriers, AS&Rs face other obstacles when accessing healthcare services. These include language barriers, personal and cultural beliefs, and financial costs [18,19,20], all of which can have an effect on the consumption of medicines [21]. Barriers in accessing medicines can affect patient safety [19] and limit adherence to treatment regimens [22]. Therefore, overcoming these barriers is necessary to improve the quality of access to medicines and to ensure that patients obtain the right choice of medicine at the right time [19]. Cultural and religious beliefs can also have an effect on access to healthcare and treatment [23]. According to the Federal Office for Migration and Refugees (BAMF), approximately 84% of Syrian nationals who applied for asylum in 2019 mentioned Islam as their religion [2]. Islam recommends avoiding the consumption of food or beverages that contain alcohol or pork products [24], both of which maybe present as active ingredients in medicines, e.g., porcine heparin, as excipients, ethanol in many pharmaceuticals, and pork gelatin in capsules [25].

This research is part of a larger study investigating the access to, and use of, medicines among Syrian AS&Rs in the German state of NRW, which has the largest population in Germany and the highest number of registered AS&Rs [2]. The aim of this study was to explore the barriers to accessing medicines among Syrian AS&Rs, and to provide an understanding of their perspectives towards taking medicines that contain alcohol or pork products.

## 2. Materials and Methods 

### 2.1. Study Design

This was a cross-sectional study using a survey for data collection. We used descriptive statistics to analyze quantitative data. Responses to open-ended survey questions were studies using thematic analysis. The qualitative data analysis supported the interpretation of the quantitative findings. 

### 2.2. Sampling Design 

Studies that focus on AS&Rs typically lack a sampling frame, which affects the possibility of using random sampling and obtaining a representative sample [26]. Hence, nonprobability sampling methods are typically used [27]. To reach a sample that can be as representative as possible, we used quota sampling in conjunction with convenience and snowball sampling. Quota sampling is accomplished by dividing a population into relevant strata [27]. In this study, we divided the population according to the key demographic variables, age and sex, which helped to balance them [28]. We estimated the fraction of each strata using the census from the statistics office of NRW, which provided information about the age and sex distribution of Syrian AS&Rs in NRW [29]. The sample was then defined using convenience and snowball sampling. In convenience sampling, researchers take a sample from a group of available, potential cases [27]. In snowball sampling, the respondents who are initially contacted are then asked to invite their social contacts within the population of interest to participate. Had we depended on convenience sampling only, we might not have reached groups of asylum seekers isolated from the rest of the community due to reasons such as being too sick to attend a school or a social event. Snowball sampling can help in overcoming this challenge and reaching isolated groups [30]. To reduce selection bias that could have resulted from snowball sampling, we used multiple entry points into the Syrian refugee community [26,30]. To ensure that our sample distribution is similar to the target population distribution along the key demographic variables, age and sex, we stopped interviewing AS&Rs who would belong to a certain strata once the pre-estimated fraction of this strata has been met. Since adequate data that report the use of medicines among refugees in Germany were not available, we were unable to perform a power calculation to determine the sample size for this study. We aimed for a relatively large sample size (1500 individuals). This was a pragmatic decision based on discussions with a biostatistician.

### 2.3. Participants and Eligibility Criteria 

#### Inclusion Criteria

AS&Rs of all ages who had their addresses registered in the state of NRW were eligible. Syrians, including stateless Palestinian Syrians and Syrian Kurds, were also eligible. In this study, the term refugee included those whose asylum applications in Germany had been approved by the BAMF, those who were entitled to refugee status or subsidiary protection, and those who were allowed to stay in Germany due to a ban on deportation.

### 2.4. Community Involvement

Conducting research in refugee populations is challenging as the population is difficult to reach and sample for a variety of reasons such as inadequate sampling frames, language or cultural issues, concerns about privacy, or fear due to experiences in home countries [31,32]. To address the challenges of participant recruitment and to enhance our understanding of the study matter, we enlisted the help of Syrian activists and other prominent members of the Syrian community in NRW who voluntarily took part in different stages of our research, including during the development of our questionnaire. They provided comments and suggestions for the questions we included and on its translation. We also asked Syrian AS&Rs to suggest places and locations where we could recruit participants for the study. In addition, many AS&Rs played the role of access points in our snowball sampling and motivated other potential participants to take part in the study. Finally, Syrian AS&Rs were asked to comment and provide feedback on the study’s findings.

### 2.5. Questionnaire Development

The questionnaire we used was primarily based on validated instruments and questions that have been applied in other studies. Information about the use of medicines was collected using questionnaires from the German Health Interview and Examination Survey for Adults (DEGS) [33], and the German Health Interview and Examination Survey for Children and Adolescents (KiGGS) [34]. Our questionnaire also included the Brief Medication Questionnaire (BMQ) [35].

Our questionnaire contained items that also asked about the barriers facing AS&Rs when accessing medicines, which was developed for this study. This part of the questionnaire was based on information from previous studies that focused on barriers to accessing healthcare services or medicines among refugees or migrants in different countries [19,20,22]. When designing this part of the questionnaire, we took into consideration the perspectives of Syrian AS&Rs. Participants suggested adding the factor ‘unavailability of certain medicines in German pharmacies’, as they mentioned that certain medicines used in Syria, or specific doses of medicines, were not available in Germany. Participants also suggested adding the possibility of purchasing medicines over the counter as another factor, as it is common to purchase some medicines (such as antibiotics) without a prescription in Syria [36], which is not possible in many European countries [37]. Both suggestions were included in our questionnaire.

Items in this part of the questionnaire were categorized into two sections. One included questions about the impact of four factors on accessing medicines: (*Based on your experience, how much do these factors limit your access to medicine in Germany?*): (1) language barriers, (2) availability of certain medicines in German pharmacies (*Finding the medicine I need in the pharmacies)*, (3) financial barriers (*I need to pay for the medicine*) and, (4) possibility of purchasing the medicines without a prescription (*The medicine I need is a prescription medicine*). Participants were asked to give their answers to each of these items on a scale from 1 to 7 (1 = No effect at all, 7 = Very large effect). The second group of questions focused on acceptance levels in regard to taking any medicine that contained alcohol or pork products. Supplementary File 1 includes the questions from this part of the questionnaire.

The remaining parts of the questionnaire included questions regarding sociodemographic and socioeconomic parameters taken from the validated instruments of different studies as well as an open-ended question that solicited comments. [38,39,40]. The questionnaire was available in three versions: one for adults (≥18), one for children (0–13) whose parents answered for them, and a version for adolescents (14–17 years old) who answered the questions themselves after the approval of one of their parents or guardians. Supplementary Table S1 provides an overview of the questionnaire.

To ensure that the questions would be culturally appropriate for our target population, we discussed the questionnaire with five Syrian refugees. We also contacted nine researchers and practitioners with work experience in the field of refugee healthcare and/or drug utilization research and asked them to check for face and content validity.

The questionnaire was developed in English. Some of the included questions underwent a validated translation to Arabic. To obtain a comprehensive Arabic version of the questionnaire we carried out the following recommended activities [41]: (1) forward translation: one of the researchers (SA), whose mother tongue is Arabic and who speaks fluent English; (2) expert panel: a panel of five experts, all of whom are native Arabic speakers and fluent in English, discussed the translated version of the questionnaire. A back translation was not possible due to limited time and resources; and (3) cognitive pre-test: we asked twelve AS&Rs to answer our questionnaire. Respondents were asked about any term or question they found unclear or possibly offensive.

In a pilot study, the questionnaire was tested in 70 participants (50 adults, 9 adolescents, and the parents of 11 children). After running the pilot study, we realized that reaching or contacting female and older participants (≤60 years of age) demanded more effort. In addition, some changes to the questionnaire were made, e.g., we changed the order of some questions so they were easier to answer. Supplementary Table S1 lists these changes.

### 2.6. Recruitment and Data Collection

One researcher (SA), who is fluent in Arabic and with a trusted relationship with the community, recruited participants for the study. The investigator visited fifteen refugee accommodation centers in the larger Cologne area. The investigator visited the accommodation centers more than once to meet the highest possible number of potential study participants. In addition to accommodation centers, the investigator visited a community center that ran a language school and consultation office for refugees three times per week, on average, during the data collection period. We recruited for this study in other places frequented by the Syrian community, including Syrian restaurants and cafes, social events for the Syrian community and at exhibitions about Syria. 

The investigator introduced himself to the participants in Arabic, informed them of the study, and explained that participation was voluntary, and that the questionnaire would not include any questions that could lead to personal identification. Data collection in this study was based on: (1) investigator-administered personal interviews about medicines use and (2) self-filled questions about demographics. We administrated a digital form of the questionnaire on tablet computers using the survey tool Qualtrics [42]. Data for this study was collected between 10 July 2019 and 31 December 2019.

### 2.7. Data Analysis

Two types of data were collected: (1) qualitative data from the answers to the open-ended question regarding access to and the use of medicines in Germany and, (2) quantitative data from the answers to the remaining questions.

#### 2.7.1. Qualitative Data Analysis

After translating the answers to the open-ended question into English, we used thematic analysis to categorize these answers in an Excel sheet (Microsoft office 2019) based on the following thematic areas that were discussed or addressed by the participants: language barriers, unavailability of certain medicines in pharmacies, financial barriers, purchasing medicines without a prescription, and acceptance of taking medicines that contain alcohol or pork products. The thematic analysis was done by the first two authors (SA, VJW). Predefined and newly emerging codes were used to categorize the data. For instance, predefined codes included pork, alcohol, and faith. Newly emerging codes were stigma and the role of family members to overcome language barriers. Triangulation between the thematic analysis and the quantitative findings were used to enhance our understanding of the nature and relevance of the access barriers described. Significant sections from participant statements were selected to be included in the results section. In addition, the Patients’ Lived Experience with Medicine model (PLEM) [43] was used to describe and summarize the aspects of participants in regard to accessing and using medicines. The PLEM module has three major themes: medicines-related burdens, medication-related beliefs, and medication-taking practice [43].

#### 2.7.2. Quantitative Data Analysis

We calculated the medians and interquartile ranges (IQRs) for the questions on barriers to medicines among adult participants and parents. To detect significant differences for each item, we conducted nonparametric analyses, as our data showed some evidence of a skewed distribution. We used the Mann–Whitney U test to examine the differences between female and male adult participants, and between participants with and without chronic diseases based on their answers to the questions regarding barriers to accessing medicines. We used the Kruskal–Wallis test to investigate differences between age groups. 

We calculated the medians and IQRs for ordinal measurement items concerning the acceptance of taking a medicine that contained alcohol or pork products. The same nonparametric analyses were applied to detect significant sex and age differences, or differences between participants with and without chronic diseases for each of those questions. A *p*-value of 0.05 was used as a cut-off value for significance in all of the tests. Analyses of quantitative data were conducted using IBM SPSS Statistics version 25 (SPSS Inc., Chicago, IL, USA).

## 3. Results

We recruited 1,641 Syrian AS&Rs. Of these, 1063 were adults (≥18 years), 456 were children (≤13 years) for whom one parent answered the questions for them, and 122 were adolescents (14–17 years). Males comprised 62.4% of the participants. Table 1 lists the sociodemographic characteristics of the participants.

**Figure 1:**
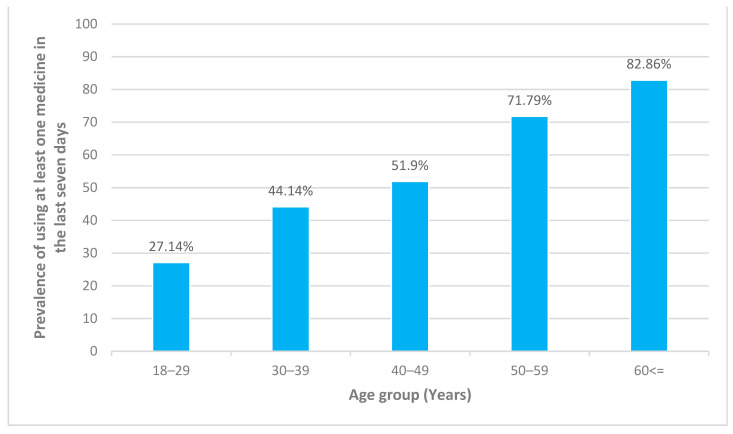
Prevalence of using at least one medicine in the last seven days among adult participants in each age group.

Approximately 41% of adult participants took at least one medicine in the last seven days before the data collection. The prevalence of taking at least one medicine increased with age (Figure 1)

### 3.1. Barriers to Accessing Medicines

Among the adult and parental respondents, language barriers had the strongest effect on accessing medicines (median 3 (2–5)), which was followed by barriers related to purchasing a medicine without a prescription (median 1 (1–3)). Financial barriers and the unavailability of certain medicines in German pharmacies had the lowest effect on the adult participants (median 1(0)) (Table 2). There was a statistically significant difference (*p* < 0.001) between adults and parental respondents concerning the effect of language barriers on access to medicines. Language barriers had a stronger impact on parents attempting to access medicines for their children in comparison to adults wanting access for themselves (Table 2). There was no statistically significant difference (*p* > 0.05) between adults and parental participants regarding the effect of the other factors that might form barriers.

**Figure 2:**
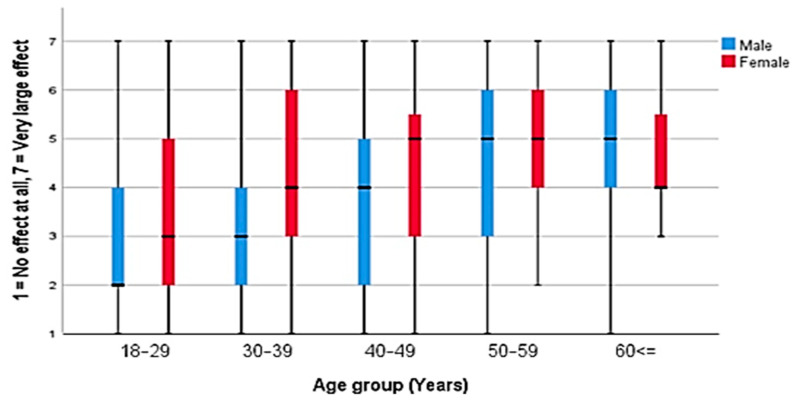
The effect of language barriers among adult participants according to sex and age.

There was a statistically significant difference (*p* < 0.001) between adult males and females in regard to the language barriers, with a stronger effect observed in females (Figure 2). There were no statistically significant differences (*p* > 0.05) between males and females regarding the other three factors (Table 2).

A significant difference (*p* < 0.05) between the different adult age groups was found in relation to access to medicines and language barriers (Figure 2), financial barriers, and purchasing medicines without prescriptions (Table 2). The effects of these three factors were stronger in those older than 50 years of age. There was no statistically significant difference (*p* > 0.05) between the different age groups concerning the unavailability of certain medicines in German pharmacies (Table 2).

There was a significant difference (*p* < 0.001) between participants with and without chronic diseases on the effect of language barriers, financial barriers, and purchasing medicines without prescription on access to medicines, with the effects of these three factors stronger in those with chronic illnesses. However, there was no statistically significant difference (*p* > 0.05) between the two groups according to the unavailability of certain medicines in German pharmacies (Table 2). 

Ninety-seven participants provided answers to the open-ended question regarding their experiences accessing and using medicines. Many of the participants stated that they had experienced no health problems since their arrival and, therefore, had never been to a doctor or a pharmacy in Germany. Hence, they were not confronted with any barriers to healthcare services in general. One participant who had multiple morbidities and took numerous medicines explained that he did not know, due to language barriers, the order in which he needed to take his medicines. Some of the participants with chronic diseases or disabilities mentioned that their health issues had reduced their ability to learn German. For example, for health reasons, they could not visit German language classes: “I started to learn the language, but I couldn’t follow because I could see only up to 15%. In the school they told me that I should go to a school for disabled people.” 

Some participants explained how they overcame language barriers by having family members accompany them to doctors or pharmacies: “Sometimes when I go to the doctor, my daughter comes with me to translate for me.” One participant suggested pharmacists hand out an Arabic version of the medicines leaflets to help overcome the language barrier.

Apart from language barriers, two participants mentioned barriers encountered when purchasing medicines without a prescription. In both cases, the participants referenced the effort needed to get an antibiotic in Germany due to the regulations regarding dispensing medicine. Two participants cited financial barriers by mentioning that medicines are expensive in Germany, especially those that are not covered by health insurance.

Many participants mentioned that their perspectives on the treatment and management of their diseases were not taken in account by physicians, due to language barriers. Some of them were not offered any treatments for what they described as serious pain or health problems and were, instead, advised to drink water. One participant described a medical error that involved administrating a medicine that caused her harm. These issues can be directly linked to language barriers. The qualitative data included also comments from participants about medications adverse events and medication routines. Some participants stated that positive beliefs and experiences with medicines were a main driver for them to adhere to medicines.

### 3.2. Acceptance of Medicines Containing Alcohol or Pork Products

Among adult participants, half of the observations concerning the acceptance of taking medicines that contain alcohol or pork products (on a scale of 1–7) were above the median value 4 (1–7). There was a statistically significant difference (*p* < 0.001) between male and female adult participants in regard to taking medicines that contain alcohol or pork products. Male participants displayed more acceptance than females (Table 3). There was a significant difference (*p* < 0.001) between the age groups as well, with those younger than 50 years old more accepting of the idea than older participants. (Table 3).

**Figure 3:**
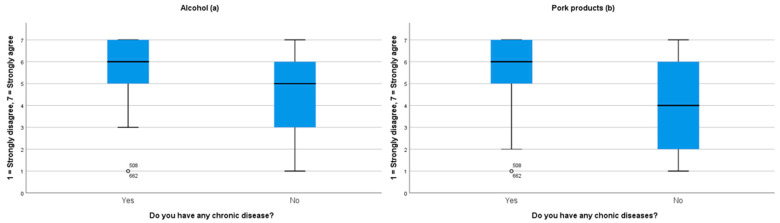
This figure represents the respondents’ answers to whether they would be willing take medication containing (**a**) alcohol or (**b**) pork products in cases when there are no other alternatives to these medicines.

There was no statistically significant difference between participants with and without chronic diseases in terms of their acceptance towards taking medicines that contain alcohol or pork products. However, there was a statistically significant difference (*p* < 0.001) between participants with and without chronic diseases regarding their acceptance of medicines that contain alcohol or products that do not have other alternatives. In this case, participants with chronic diseases showed more acceptance of medicines that contain alcohol or pork products (Figure 3). We also observed that some participant subgroups showed more acceptance towards taking a medicine with alcohol than with pork products (Table 3).

An analysis of the qualitative data provided explanations for some of the perspectives concerning the acceptance of taking medicines that contain alcohol or pork products. Many of the participants suggested that they would take these medicines if there was no other alternative, as it would be for medical reasons: “If a medicine has no alternative that is free of alcohol or pork products, I would take it then. God did not ask us to do the impossible.” Other participants explained why they would not think to check whether a medicine contained these products. They suggested that it was difficult to check the ingredients of medicines due to language barriers, and that it did not occur to them that medicines would contain these products, as they are not used to this in Syria.

## 4. Discussion

To the best of our knowledge, this is the first study to investigate and report on the barriers encountered when accessing medicines among a group of AS&Rs in Germany. Our results fill important knowledge gaps and support the development of recommendations to improve access to medicines for AS&Rs. We found that language barriers formed the major obstacle compared to the other three barriers we considered. Other studies have also reported that language barriers usually affect access to medicines among different groups of refugees in different countries [19,20,45]. For example, in a study from England, researchers found that refugee parents considered language barriers to be a major factor that limited their access to medicines for their children [46]. Language barriers were followed by barriers related to obtaining medicines without a prescription, financial barriers, and the unavailability of certain medicines in German pharmacies. The qualitative data illustrated the challenge related to obtaining medicines without a prescription by highlighting the difficulty in obtaining certain medicines, such as antibiotics, without a prescription in Germany. It is simple to get these medicines without a prescription in Syria [37]. Financial barriers and the unavailability of certain medicines in German pharmacies both played minor roles in limiting access. This can be explained by the fact that 98% of the participants were covered by regular health insurance. It is noteworthy, that this is not the financial burden for the individuals because the health system is financing medicines. Germany has a high level of public spending on health. Out-of-pocket expenditure on health is only 12% in Germany. As the results of this study show, financial burden was not found to be a relevant barrier. This is very different from many other countries in which medicines is entirely or almost entirely financed by individuals [47]. A study that focused on barriers to accessing healthcare services among refugees in Austria also reported minor effects due to financial barriers [48]. 

Our results showed a statistically significant difference between females and males concerning the effect of language barriers on access to medicines, with the effect being stronger and more limiting for females. In Germany, female refugees attend integration courses less frequently and with longer delays after their arrival in Germany in comparison to males. Female refugees also assessed their German language skills as weaker than males do. This may be due to female refugees having less opportunities for using or practicing the German language. Female refugees who come to Germany with families tend to focus on housing provision and childcare and would, therefore, have less time to learn German [49]. This is not a new phenomenon. Studies on refugees who arrived in Germany in the nineties also showed a slower integration of female refugees compared to their male counterparts [49,50].

There was a statistically significant difference between participants from different age groups according to the effects of language barriers, financial barriers, and the possibility of purchasing a medicine without a prescription. The impact of these three factors on limiting access to medicines was strongest among participants who were older than 50 years of age. Older age is associated with a higher risk of chronic diseases and higher medical referral rates [20]. One study has shown that older refugees had lower levels of education, higher unemployment rates, and poorer language skills of the hosting countries in comparison to younger refugees [51]. This could explain why the effect of these factors was strongest among older participants. Our qualitative analysis also demonstrated that some young respondents, due to their good general health status, seldom acquired any medicines after their arrival to Germany. Hence, they were rarely confronted with the process of trying to get prescription medicine.

There was also a statistically significant difference between those with and without chronic diseases according to language barriers, financial barriers, and the possibility of purchasing medicines without a prescription. Our qualitative data analysis indicated that some participants with chronic diseases or disabilities faced reduced opportunities for learning German due to their health issues. Treating chronic diseases usually requires the long-term use of medicines [23]. Many of these medicines can only be purchased with a prescription and would require co-payments from the patients. This may be why access to medicines in this group was more strongly influenced by language barriers, financial barriers, and the possibility of purchasing a medicine without a prescription. 

Ninety-seven participants provided comments about their perspectives or experiences related to medicines access and use in Germany. The answers to the open-ended question did not represent the total sample but they provided an overview of the experiences or perspectives of these ninety-seven participants regarding medicines access and use. Why many participants did not provide an answer to this question, is unclear but it is likely that they did not have comments regarding medicines access and use. Our qualitative analysis illustrated some of the strategies participants have used to overcome language barriers. For example, some mentioned that family members would accompany them to doctor appointments to translate for them. While this might be a practical solution, there is an important ethical issue associated with using an untrained person to interpret health information, and with exposing a family member, especially children, to information regarding traumatic experiences [22]. One participant suggested preparing medicine leaflets in Arabic. This could be an effective and feasible solution that could help many individuals overcome some of the language barriers they encounter, as the Arabic literacy rate among Syrian nationals is high (86.4%) [52].

The qualitative data also included comments from participants about how their perspectives about their health conditions were not taken into account by healthcare professionals. This led to ineffective treatment for their health conditions. According to the World Health Organization’s report, Medication Without Harm patients are generally “passive recipients” of medicines and are often not involved in the process of the treatment or management of their diseases [53]. However, taking into consideration patient perspectives are major contributors to a successful treatment [54]. Refugees might be more prone to being left out of the treatment decision-making process due to language barriers. A participant mentioned that she, due to a medical error, applied a medicine that caused her harm. Medicines sometimes have complex names or packaging, and may lack sufficient or clear information. Confusing medicines names, labeling, and packaging are frequent sources of error [53]. AS&Rs can also be more prone to these errors due to language barriers. Providing a translated version of a medicine’s leaflet to patients who ask for it could be helpful in reducing the possibility of medical errors. More research is needed to identify the demand and need for information on medicines, their risks (e.g., antimicrobial resistance) and benefits in this population. 

While developing the questionnaire for our study, Syrian AS&Rs suggested adding the challenge of purchasing a medicine without a prescription as a barrier to accessing medicines. Participants in this study considered purchasing medicines without a prescription as the second most important factor to limit access to medicines after language barriers. Restricting the dispensing of antibiotics to prescription-only is recommended to promote the adequate use of medicines and prevent increases in antimicrobial resistance [37]. 

There is a lack of information concerning refugees’ acceptance of ‘Western medicine’ [22]. In our study, we presented the perspectives of a group of adult Syrian AS&Rs on their acceptance towards taking medicines that contain alcohol or pork products. Accepting the idea of taking these medicines is an issue, not only for Muslim patients, but also for those of other religions such as Jews, or those who follow certain diets (vegans) [25].

We found a significant difference between female and male participants regarding their acceptance towards taking medicines that contain alcohol or pork products, with females being less accepting than males. There was also a significant difference between age groups. Those who were younger than 50 years of age showed more acceptance than those who were older. We do not have an explanation for these results and suggest further qualitative studies are needed to understand them.

There was no statistically significant difference between those with and without chronic diseases in terms of their acceptance towards taking medicines that contain alcohol or pork products. However, those with chronic diseases were significantly more accepting of these medicines in instances where there was no other alternative to the medicines. Our qualitative analysis illustrated the opinions of some participants and showed that if there were no alternatives, then taking these medicines should be tolerated. One study has suggested that Muslims can take medicines that contain alcohol or pork products if there is no alternative and in cases of emergency [55].

Our participants showed more acceptance towards taking medicines that contain alcohol than ones that contain pork products. Some Muslim countries accept or allow the use of medicines that contain alcohol (benzyl alcohol, methyl alcohol, and polyethylene alcohol) [56]. However, this does not apply to those that contain pork products. Some schools of Islamic jurisprudence refuse to accept the use of medicines that contain pig gelatin even though it is highly purified and so degraded that the pig DNA cannot be detected, nor can the original source of the gelatin be identified [57].

The results of this study help to understand the barriers to accessing medicines among Syrian AS&Rs not only in NRW, but in other parts of Germany as well. Barriers to accessing medicines among Syrian AS&Rs might have been different, or had different effects, if we had compared them to other groups of refugees from different nationalities. Those who are granted a refugee status have access to free language classes and the right to regular health insurance [12], which can reduce the impact of barriers to medicines or healthcare in general. The approval quota for staying in Germany among Syrians who have applied for asylum in Germany is 83.7%, which is higher than for asylum seekers from Iraq (35%) or Afghanistan (38%) [2]. This means that the impact of these barriers could be higher among those groups if they were to be compared to Syrian AS&Rs. 

### Strengths and Limitations

Finally, a number of important limitations need to be considered. Due to the lack of a sample frame, we used non-probability sampling methods. This can affect the possibility of the representativeness of our results to the Syrian AS&Rs population in NRW or in greater Germany. Nevertheless, given that there is no data available that would allow for drawing a representative sample, this is an important first step towards understanding medicine access barriers among Syrian AS&Rs in Germany. To enhance the likelihood of our sample being representative, we used a combination of sampling methods and recruited study participants from a wide range of settings that are typical to our study population. We also aimed for, and achieved, a large sample size that included a diverse group of AS&Rs. Another strength of our study is that we involved our target population in several stages of the research. This was helpful in ensuring that our investigation was well-adapted to the culture of the target population. The qualitative analysis complemented the quantitative analysis and provided a deeper understanding and explanation for how certain barriers affect Syrian asylum seekers and refugees’ access to medicine, and their acceptance towards taking medicines that contain alcohol or pork products. However, there are still many knowledge gaps about medicines access in AS&R in Germany. For instance, more qualitative research is needed to provide a better understanding for AS&Rs perspectives towards accessing and using medicines. To study medicines use among specific age groups of AS&Rs, future studies should consider larger sample sizes.

## 5. Conclusions

Optimizing the access to and use of medicines to enhance the quality of health care for refugees has global relevance. Overcoming the barriers facing refugees in accessing medicines is necessary for guaranteeing their quality use [19]. Based on our results, we recommend applying more effort to enhancing the ability of AS&Rs to learn the German language as a mid–to long-term strategy to overcome language barriers. Particular focus should be placed on female refugees, older refugees, and those with chronic diseases or disabilities. We also recommend providing translated medical leaflets for patients who ask for them and medical interpreting services. This would help them to understand information regarding the medicines they take, including whether or not these medicines contain alcohol or pork products. Having pharmacy staff demonstrate effective cross-cultural communication skills would also be helpful in addressing barriers and any other cultural issues that could be related to the access and use of medicines [22]. The barriers we have addressed in our study may also affect other cultural and linguistic minorities in diverse communities and not just refugees [22]. Overcoming these barriers requires the provision of more training and education that takes into consideration cross-cultural factors, not only for refugees, but also for physicians, pharmacists, and their staff [45]. Healthcare professionals should try to have a better understanding of the conditions of asylum seeker and refugee patients and recognize their perspectives on their treatments and diseases. This is crucial for any successful intervention among patients [54]. There is a need for studies that address other factors that might limit AS&Rs from accessing healthcare services in general and medicines in Germany; this includes cultural barriers and discrimination in healthcare.

## Figures and Tables

**Table 1:** Sociodemographic characteristics of the study participants.

Participant Characteristics	Number of Participants	Proportion (%)
**Sex**		
Male	1024	62.4%
Female	617	37.6%
**Age**		
0–17	578	35.22%
18–29	468	28.52%
30–39	318	19.38%
40–49	158	9.63%
50–59	78	4.75%
≥60	41	2.5%
**Health insurance**		
Yes	1616	98.48%
No	23	1.4%
Missing	2	0.12%
**Residency status**		
Refugee status or subsidiary protection	1603	97.68%
Asylum seeker	38	2.32%
**Accommodation**		
Initial reception center	18	1.1%
Long-term accommodation center	318	19.38%
Private housing	1289	78.55%
Missing	16	0.97%
**Chronic disease (Adults only)**		
Yes (22 different chronic diseases)	146	13.74%
No	914	85.98%
Missing	3	0.28%
**The most common chronic diseases**		
Hypertension	39	26.71%
Diabetes	26	17.81%
Hypothyroidism	18	12.33%
Hyperlipoproteinemia	6	4.11%
Migraine	5	3.42%
Other chronic diseases	52	35.62%
**Employment (Adults only)**		
Employed	249	23.42%
Retired	16	1.51%
Vocational training	237	22.29%
Not employed	537	50.52%
Missing	24	2.26%
**German language level ^1^**		
A1-A1	242	22.77%
B1-B2	495	46.57%
C1-C2	168	15.8%
None	131	12.32%
Missing	27	2.54%
**Marriage status (Adults only)**		
Single	381	35.84%
Married	613	57.67%
In a relationship	27	2.54%
Divorced	19	1.79%
Widowed	13	1.22%
Missing	10	0.94%

^1^ Language levels were classified according to the Common European Framework of Reference for Languages (CEFR) [44].

**Table 2:** Descriptive statistics for different participant groups according to four factors influencing access to medicines.

Participant Subgroups			Language Barriers	Unavailability of Certain Medicines in Pharmacies	Financial Barriers	Purchasing Medicines without a Prescription
**Total ^1^**						
	Adults	Median (IQR ^2^)	3 (2–5) ***	1 (0)	1 (0)	1 (1–3)
	Parents	Median (IQR)	4 (3–5) ***	1 (0)	1 (0)	1 (1–3)
**Adults**						
**Sex ^1^**						
	Female	Median (IQR)	4 (2–5) ***	1 (0)	1 (0)	1 (1–3)
	Male	Median (IQR)	3 (2–4) ***	1 (0)	1 (0)	1 (1–3)
**Age ^3^**						
	18–29	Median (IQR)	2 (2–4) ***	1 (0)	1 (0) **	1 (0) *
	30–39	Median (IQR)	4 (2–5) ***	1 (0)	1 (0) **	1 (1–3) *
	40–49	Median (IQR)	4 (3–5) ***	1 (0)	1 (0) **	1 (1–3) *
	50–59	Median (IQR)	5 (4–6) ***	1 (0)	1 (0) **	1 (1–3) *
	≤60	Median (IQR)	5 (4–6) ***	1 (0)	1 (1–3) **	1 (1–4) *
**Chronic diseases ^1^**						
	Yes	Median (IQR)	4 (3–5) ***	1 (0)	1 (1–2) ***	1 (1–3) ***
	No	Median (IQR)	3 (2–5) ***	1 (0)	1 (0) ***	1 (1–2) ***

^1^ Mann–Whitney U test. ^2^ Interquartile range. ^3^ Kruskal–Wallis ANOVA Test. * Difference is significant at the 0.05 level. ** Difference is significant at the 0.01 level. *** Difference is significant at the 0.001 level.

**Table 3:** Descriptive statistics for different participant subgroups concerning their acceptance of taking medicines that contain alcohol or pork products.

Participant Subgroups			Alcohol	Pork Products
**Total**				
	Adults	Median (IQR ^1^)	4 (1–7)	4 (1–7)
**Sex ^2^**				
	Female	Median (IQR)	1 (1–4) ***	1 (1–4) ***
				
	Male	Median (IQR)	7 (3–7) ***	4 (2–7) ***
**Age ^3^**				
	18–19	Median (IQR)	7 (1–7) ***	7 (1–7) ***
	30–39	Median (IQR)	4 (1–7) ***	4 (1–7) ***
	40–49	Median (IQR)	4 (1–7) ***	3 (1–7) ***
	50–59	Median (IQR)	4 (1–7) ***	4 (1–7) ***
	≥60	Median (IQR)	4 (1–5) ***	4 (1–4) ***
**Chronic diseases ^2^**				
	Yes	Median (IQR)	4 (2–7)	4 (1–7)
	No	Median (IQR)	4 (1–7)	4 (1–4)

^1^ Interquartile range. ^2^ Mann–Whitney U test. ^3^ Kruskal–Wallis ANOVA Test. *** Difference is significant at the 0.001 level.

## Data Availability

Data available on request due to restrictions e.g., privacy or ethical. The data presented in this study are available on request from the corresponding author.

## Supplementary Materials

The following supplementary materials are available online at https://www.mdpi.com/1660-4601/18/2/519/s1, Table S1: An overview of the questionnaire, Supplementary File 1: Questions from the part of the questionnaire that focuses on barriers to accessing medicines among Syrian asylum seekers and refugees.

## References

[1] United Nations High Commissioner for Refugees Global Trends Forced Displacement in 2019. https://www.unhcr.org/5ee200e37.pdf.

[2] Bundesamt für Migration und Flüchtlinge Das Bundesamt in Zahlen 2019: Asyl, Migration und Integration, 2020. https://www.bamf.de/SharedDocs/Anlagen/DE/Statistik/BundesamtinZahlen/bundesamt-in-zahlen-2019.pdf?__blob=publicationFile&v=4.

[3] Gottlieb N., Bozorgmehr K., Trummer U., Rechel B. (2019). Health policies and mixed migration—Lessons learnt from the ‘Refugee Crisis’. Health Policy.

[4] Puchner K., Karamagioli E., Pikouli A., Tsiamis C., Kalogeropoulos A., Kakalou E., Pavlidou E., Pikoulis E. (2018). Time to rethink refugee and migrant health in Europe: Moving from emergency response to integrated and individualized health care provision for migrants and refugees. Int. J. Environ. Res. Public Health.

[5] United Nations High Commissioner for Refugees Global Report, 2005. https://www.unhcr.org/449267670.pdf.

[6] United Nations High Commissioner for Refugees The 1951 Refugee Convention. https://www.unhcr.org/1951-refugee-convention.html.

[7] Bozorgmehr K., Samuilova M., Petrova-Benedict R., Girardi E., Piselli P., Kentikelenis A. (2019). Infectious disease health services for refugees and asylum seekers during a time of crisis: A scoping study of six European Union countries. Health Policy.

[8] Bozorgmehr K., Razum O. (2015). Effect of restricting access to health care on health expenditures among asylum-seekers and refugees: A quasi-experimental study in Germany, 1994–2013. PLoS ONE.

[9] Bozorgmehr K., Razum O. (2016). Refugees in Germany—Untenable restrictions to health care. Lancet.

[10] Bozorgmehr K., Wenner J., Razum O. (2017). Restricted access to health care for asylum-seekers: Applying a human rights lens to the argument of resource constraints. Eur. J. Public Health.

[11] Bundesministerium für Gesundheit Zuzahlung und Erstattung von Arzneimitteln. https://www.bundesgesundheitsministerium.de/zuzahlung-und-erstattung-arzneimittel.html#c12485.

[12] Bundesamt für Justiz Asylbewerberleistungsgesetz (AsylbLG). https://www.gesetze-im-internet.de/asylblg/BJNR107410993.html.

[13] OECD European Union (2018). Health at a Glance: Europe 2018. State of Health in the EU Cycle.

[14] Bauhoff S., Göpffarth D. (2018). Asylum-seekers in Germany differ from regularly insured in their morbidity, utilizations and costs of care. PLoS ONE.

[15] Claassen K., Jäger P. (2018). Impact of the introduction of the electronic health insurance card on the use of medical services by asylum seekers in Germany. Int. J. Environ. Res. Public Health.

[16] Epping B. (2017). Medizinische Versorgung von Flüchtlingen: Teure Hürden. Z. Orthop. Unfall..

[17] §264 SGB V—Übernahme der Krankenbehandlung für Nicht Versicherungspflichtige Gegen Kostenerstattung. http://www.lexsoft.de/cgi-bin/lexsoft/justizportal_nrw.cgi?xid=137489,351.

[18] Jäger P., Claassen K., Ott N., Brand A. (2019). Does the electronic health card for asylum seekers lead to an excessive use of the health system? Results of a survey in two municipalities of the German ruhr area. Int. J. Environ. Res. Public Health.

[19] Kay M., Wijayanayaka S., Cook H., Hollingworth S. (2016). Understanding quality use of medicines in refugee communities in Australian primary care: A qualitative study. Br. J. Gen. Pract..

[20] Hadgkiss E.J., Renzaho A.M.N. (2014). The physical health status, service utilisation and barriers to accessing care for asylum seekers residing in the community: A systematic review of the literature. Aust. Health Rev.

[21] Lerner-Geva L., Blumstein T., Boyko V., Farhi A., Benyamini Y. (2017). Cultural disparities in the use of prescription and nonprescription medications among midlife women in Israel. Int. J. Health Serv..

[22] Bellamy K., Ostini R., Martini N., Kairuz T. (2015). Access to medication and pharmacy services for resettled refugees: A systematic review. Aust. J. Prim. Health.

[23] Shahin W., Kennedy G.A., Stupans I. (2019). The impact of personal and cultural beliefs on medication adherence of patients with chronic illnesses: A systematic review. Patient Pref. Adherence.

[24] Attum B., Hafiz S., Malik A., Shamoon Z. (2020). Cultural Competence in the Care of Muslim Patients and Their Families.

[25] Bruhn C. (2016). Koscher, Halal und Vegan: Was Ist Verboten, Was Ist Erlaubt?. Deutsche Apotheker Zeitung.

[26] Bloch A. (2007). Methodological challenges for national and multi-sited comparative survey research. J. Refug. Stud..

[27] Enticott J.C., Shawyer F., Vasi S., Buck K., Cheng I.-H., Russell G., Kakuma R., Minas H., Meadows G. (2017). A systematic review of studies with a representative sample of refugees and asylum seekers living in the community for participation in mental health research. BMC Med. Res. Methodol..

[28] Dean J., Wollin J., Stewart D., Debattista J., Mitchell M. (2012). Hidden yet visible: Methodological challenges researching sexual health in Sudanese refugee communities. Cult. Health Sex.

[29] Landesbetrieb IT.NRW Statistik und IT-Dienstleistungen. https://www.it.nrw/.

[30] Sulaiman-Hill C.M.R., Thompson S. (2011). Sampling challenges in a study examining refugee resettlement. BMC Int. Health Hum. Rights.

[31] Spring M., Westermeyer J., Halcon L., Savik K., Robertson C., Johnson D.R., Butcher J.N., Jaranson J. (2003). Sampling in difficult to access refugee and immigrant communities. J. Nerv. Ment. Dis..

[32] Zeisler M.-L., Bilgic L., Schumann M., Wengler A., Lemcke J., Gößwald A., Lampert T., Santos-Hovener C., Schmich P. (2020). Interventions to increase the reachability of migrants in Germany with health interview surveys: Mixed-mode feasibility study. JMIR Form. Res..

[33] Robert Koch Institute Studie zur Gesundheit Erwachsener in Deutschland (DEGS). https://www.degs-studie.de/deutsch/home.html.

[34] Robert Koch Institute Studie zur Gesundheit von Kindern und Jugendlichen in Deutschland (KIGGS). https://www.kiggs-studie.de/deutsch/home.html.

[35] Svarstad B.L., Chewning B.A., Sleath B.L., Claesson C. (1999). The brief medication questionnaire: A tool for screening patient adherence and barriers to adherence. Patient Educ. Couns..

[36] Al-Faham Z., Habboub G., Takriti F. (2011). The sale of antibiotics without prescription in pharmacies in Damascus, Syria. J. Infect. Dev. Ctries.

[37] Morgan D.J., Okeke I.N., Laxminarayan R., Perencevich E.N., Weisenberg S. (2011). Non-prescription antimicrobial use worldwide: A systematic review. Lancet Infect Dis.

[38] Institut für Medizinmanagement und Gesundheitswissenschaften, Universität Bayreuth Auswirkungen des Zustroms von Asylbewerbern auf Die Gesundheitliche Versorgung in Bayern. https://www.lgl.bayern.de/gesundheit/gesundheitsversorgung/doc/kurzbericht_auswirkungen_zustrom_asylbewerber_gv_by.pdf.

[39] Lampert T., Müters S., Stolzenberg H., Kroll L.E. (2014). Messung des sozioökonomischen Status in der KiGGS-Studie Erste Folgebefragung (KiGGS Welle 1). Bundesgesundheitsblatt Gesundh. Gesundh..

[40] Lampert T., Kroll L., Müters S., Stolzenberg H. (2013). Messung des sozioökonomischen Status in der Studie zur Gesundheit Erwachsener in Deutschland (DEGS1). Bundesgesundheitsblatt Gesundh. Gesundh..

[41] World Health Organization Process of Translation and Adaptation of Instruments. https://www.who.int/substance_abuse/research_tools/translation/en/.

[42] Qualtrics Provo, UT, USA, 2020. https://www.qualtrics.com.

[43] Mohammad A.M., Moles R.J., Chen T.F. (2016). Medication-related burden and patients’ lived experience with medicine: A systematic review and metasynthesis of qualitative studies. BMJ Open.

[44] Council of Europe Common European Framework of Reference for Languages (CEFR). https://www.coe.int/en/web/common-european-framework-reference-languages/level-descriptions.

[45] Clark A., Gilbert A., Rao D., Kerr L. (2014). ‘Excuse me, do any of you ladies speak English?’ Perspectives of refugee women living in South Australia: Barriers to accessing primary health care and achieving the Quality Use of Medicines. Aust. J. Prim. Health.

[46] Alkahtani S., Cherrill J., Millward C., Grayson K., Hilliam R., Sammons H., Choonara I. (2014). Access to medicines by child refugees in the East Midlands region of England: A cross-sectional study. BMJ Open.

[47] Siegel M., Busse R. (2018). Can People Afford to Pay for Health Care? New Evidence on Financial Protection in Germany.

[48] Kohlenberger J., Buber-Ennser I., Rengs B., Leitner S., Landesmann M. (2019). Barriers to health care access and service utilization of refugees in Austria: Evidence from a cross-sectional survey. Health Policy.

[49] Worbs S., Baraulina T. Geflüchtete Frauen in Deutschland: Sprache, Bildung und Arbeitsmarkt. BAMF-Kurzanalyse. https://www.bamf.de/SharedDocs/Anlagen/DE/Forschung/Kurzanalysen/kurzanalyse7_gefluchetete-frauen.pdf?__blob=publicationFile&v=14.

[50] Eisnecker P., Giesecke J., Kroh M., Liebau E., Marcus J., Salikutluk Z., Schacht D., Spieß C.K., Westermaier F. (2016). Die integration geflüchteter—Erkenntnisse aus der vergangenhei. DIW-Wochenbericht.

[51] Li W.W. (2016). Comparative study on social-economic status, trauma and mental health disorders among older and younger refugees in Australia. J. Trop. Psych..

[52] Al Hassan M., Bengtsson S., Kohlenberger J. Understanding the Syrian Educational System in a Context of Crisis. Vienna Institute of Demography Working Papers, No. 09/2016, Austrian Academy of Sciences (ÖAW), Vienna Institute of Demography (VID), Vienna, 2016. https://www.econstor.eu/bitstream/10419/156317/1/875728065.pdf.

[53] World Health Organization Medication Without Harm, WHO Global Patient Safety Challenge, 2017. https://apps.who.int/iris/bitstream/handle/10665/255263/WHO-HIS-SDS-2017.6-eng.pdf;jsessionid=80005B1CD84D740DA575470D29021F89?sequence=1.

[54] Wettermark B., Godman B., Bennie M. (2016). Drug Utilization Research: Methods and Applications.

[55] Saha T., Rifat T., Shimanto S. (2019). Prospects of halal pharmaceuticals. Asian J. Ethnopharmacol. Med. Foods.

[56] Asmak A., Fatimah S., Huzaimah I., Khuriah A.H., Siti Khadijah A.M. (2015). Is our medicine lawful (Halal)?. Middle East J. Sci. Res..

[57] Public Health England The Children’s Flu Vaccination Programme, the Nasal Flu Vaccine Fluenzand Porcine Gelatine, 2014. https://assets.publishing.service.gov.uk/government/uploads/system/uploads/attachment_data/file/386842/2902998_PHE_FluPorcine_QAforParents_FINAL_CT.pdf.

