# Correction: Aljadeeah, S., et al. Barriers to Accessing Medicines among Syrian Asylum Seekers and Refugees in a German Federal State. *Int. J. Environ. Res. Public Health* 2021, *18*, 519

**DOI:** 10.3390/ijerph18052464

**Published:** 2021-03-03

**Authors:** Saleh Aljadeeah, Veronika J. Wirtz, Eckhard Nagel

**Affiliations:** 1 Institute of Medical Management and Health Sciences, University of Bayreuth, Prieserstr. 2, 95440 Bayreuth, Germany; eckhard.nagel@uni-bayreuth.de; 2 Department of Global Health, Boston University School of Public Health, Boston, MA 02118, USA; vwirtz@bu.edu

## Error in Table

In the original article [1], there was an error in the published version of Table 1 (page 7), which included a description of the frequency and prevalence of the five most common chronic diseases among adult participants in our study. We mistakenly underestimated the frequency of some chronic diseases because we neglected to count the chronic diseases that led to the use of some of the medicines. We have now corrected the error. The corrected Table 1 appears below. The authors apologize for any inconvenience caused and state that the scientific conclusions are unaffected. The original article has been updated.

## Figures and Tables

**Table 1:** Sociodemographic characteristics of the study participants.

Participant Characteristics	Number of Participants	Proportion (%)
**Sex**		
Male	1024	62.4%
Female	617	37.6%
**Age**		
0–17	578	35.22%
18–29	468	28.52%
30–39	318	19.38%
40–49	158	9.63%
50–59	78	4.75%
60≤	41	2.5%
**Health insurance**		
Yes	1616	98.48%
No	23	1.4%
Missing	2	0.12%
**Residency status**		
Refugee status or subsidiary protection	1603	97.68%
Asylum seeker	38	2.32%
**Accommodation**		
Initial reception center	18	1.1%
Long-term accommodation center	318	19.38%
Private housing	1289	78.55%
Missing	16	0.97%
**Chronic disease (Adults only)**		
Yes (22 different chronic diseases)	146	13.74%
No	914	85.98%
Missing	3	0.28%
**The most common chronic diseases**		
Hypertension	71	6.68
Diabetes	44	4.14
Hypothyroidism	30	2.82
Hyperlipoproteinemia	21	1.98
Anemia	21	1.98
Other chronic diseases	66	6.21
**Employment (Adults only)**		
Employed	249	23.42%
Retired	16	1.51%
Vocational training	237	22.29%
Not employed	537	50.52%
Missing	24	2.26%
**German language level ^1^**		
A1-A1	242	22.77%
B1-B2	495	46.57%
C1-C2	168	15.8%
None	131	12.32%
Missing	27	2.54%
**Marriage status (Adults only)**		
Single	381	35.84%
Married	613	57.67%
In a relationship	27	2.54%
Divorced	19	1.79%
Widowed	13	1.22%
Missing	10	0.94%

^1^ Language levels were classified according to the Common European Framework of Reference for Languages (CEFR) [2].
