# Supplementary material for: Barriers to Accessing Medicines among Syrian Asylum Seekers and Refugees in a German Federal State
Source: Int J Environ Res Public Health. 2021 Jan 10;18(2):519. doi: 10.3390/ijerph18020519 (PMC7827681; doi:10.3390/ijerph18020519)
Supplement: Supplementary file 1 [file ijerph-18-00519-s001.pdf]

Table S1: An overview of the questionnaire.

Construct	Instrument	Questionnaire version	Modified or original	Type of modification	Existing or new Arabic translation
**Use of medicine**	DEGS<br>KIGGS	Adults (DEGS)<br>Children and adolescent (KiGGS)	Modified	Removed a question (about contraceptive pill intake) *<br>Added specifications and changed the order	No
**Adherence to medication for chronic diseases**	Brief Medication Questionnaire	Adults	Original	_	No
**Barriers to accessing medicine (factors)**	Self-constructed	Adults and parents	Modified	Added options<br>Simplification	_
**Acceptance of taking medicine that contains alcohol or pork products**	Self-Constructed	Adults	Modified	Simplification	_
**Socioeconomic and sociodemographic factors**	DEGS, KIGGS, REFMED	Adults, children, and adolescents	Modified	Options for some questions added.<br>A question was removed.<br>Options reduced	Yes

* Participants suggested removing a question directed to female participants concerning whether they took contraceptive pills. This question could be viewed as inappropriate and can result in scepticism and an interruption of the survey process.

Supplementary File 1: Questions from the part of the questionnaire that focuses on barriers to accessing medicines among Syrian asylum seekers and refugees.

Based on your experience, how much do these factors limit your access to medicine in Germany?

	No effect at all	2	3	4	5	6	Very large effect
Language barriers	○	○	○	○	○	○	○
Finding the medicine I need in the pharmacies	○	○	○	○	○	○	○
I need to pay for the medicine	○	○	○	○	○	○	○
The medicine I need is a prescription medicine	○	○	○	○	○	○	○

Before taking any medicine, do you check whether it contains any alcohol or pork products?

	No, never	2	3	4	5	6	Yes, every time
Alcohol	○	○	○	○	○	○	○
Pork products	○	○	○	○	○	○	○

Would you take a medicine that contains any alcohol or pork products?

	No, never	2	3	4	5	6	Yes, definitely
Alcohol	○	○	○	○	○	○	○
Pork products	○	○	○	○	○	○	○

If the medicine I need would contain alcohol or pork products and there were no alternative to it, I will take this medicine:

	Strongly disagree	2	3	4	5	6	Strongly agree
Alcohol	○	○	○	○	○	○	○
Pork products	○	○	○	○	○	○	○
